# Etymologia: *Granulicatella*

**DOI:** 10.3201/eid2409.ET2409

**Published:** 2018-09

**Authors:** Ronnie Henry

**Keywords:** etymologia, Granulicatella, bacteria, nutritionally variant streptococci, satellite streptococci, Abiotrophia, sulfhydryl compounds

## *Granulicatella* [granʹyoo-lik-ə-telʺə]

In 1961, Frenkel and Hirsch described strains of streptococci isolated from cases of bacterial endocarditis that grew only in the presence of other bacteria, around which they formed satellite colonies, or in media enriched with sulfhydryl compounds, such as cysteine. These nutritionally variant streptococci were eventually assigned the species *Streptococcus defectivus* (Latin for “deficient”) and *S. adjacens* (because it grows adjacent to other bacteria) (Figure).

**Figure:**
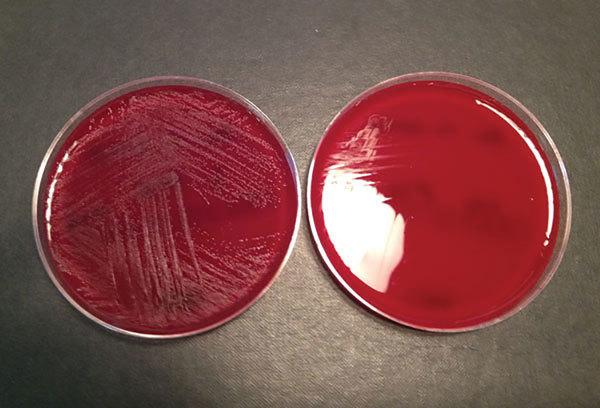
Blood agar plates with (left) and without (right) pyridoxal supplement from a study of neonatal *Granulicatella elegans* bacteremia, London, UK. Image from Neonatal *Granulicatella elegans* Bacteremia, London, UK; Emerging Infectious Diseases Vol. 19, no. 7, July 2013.

On the basis of later research, these were placed in a new genus *Abiotrophia* (Greek *a*, “un-,” + *bios*, “life,” + *trophe*, “nutrition”) as *A. adiacens* and *A. defectiva*. In 1998 and 1999, 2 additional species of *Abiotrophia* were described, *A. elegans* (Latin, “fastidious,” referring to fastidious growth requirements) and *A. balaenopterae* (isolated from a minke whale [*Balaenoptera acutorostrata*]). In 2000, these new species, along with *A. adiacens*, were reclassified in the new genus *Granulicatella* (Latin *granulum*, “small grain,” + *catella*, “small chain”).
